# Enzymes-Assisted Extraction of Plants for Sustainable and Functional Applications

**DOI:** 10.3390/ijms23042359

**Published:** 2022-02-21

**Authors:** Paulina Streimikyte, Pranas Viskelis, Jonas Viskelis

**Affiliations:** Lithuanian Research Centre for Agriculture and Forestry, Institute of Horticulture, 54333 Babtai, Lithuania

**Keywords:** enzyme-assisted extraction, plant material, phenolic compounds, oligosaccharides, prebiotic, nanocellulose, nanofibers, fermentation, sustainability

## Abstract

The scientific community and industrial companies have discovered significant enzyme applications to plant material. This rise imparts to changing consumers’ demands while searching for ‘clean label’ food products, boosting the immune system, uprising resistance to bacterial and fungal diseases, and climate change challenges. First, enzymes were used for enhancing production yield with mild and not hazardous applications. However, enzyme specificity, activity, plant origin and characteristics, ratio, and extraction conditions differ depending on the goal. As a result, researchers have gained interest in enzymes’ ability to cleave specific bonds of macroelements and release bioactive compounds by enhancing value and creating novel derivatives in plant extracts. The extract is enriched with reducing sugars, phenolic content, and peptides by disrupting lignocellulose and releasing compounds from the cell wall and cytosolic. Nonetheless, depolymerizing carbohydrates and using specific enzymes form and release various saccharides lengths. The latest studies show that oligosaccharides released and formed by enzymes have a high potential to be slowly digestible starches (SDS) and possibly be labeled as prebiotics. Additionally, they excel in new technological, organoleptic, and physicochemical properties. Released novel derivatives and phenolic compounds have a significant role in human and animal health and gut-microbiota interactions, affecting many metabolic pathways. The latest studies have contributed to enzyme-modified extracts and products used for functional, fermented products development and sustainable processes: in particular, nanocellulose, nanocrystals, nanoparticles green synthesis with drug delivery, wound healing, and antimicrobial properties. Even so, enzymes’ incorporation into processes has limitations and is regulated by national and international levels.

## 1. Introduction

The demand for new and natural compounds, ‘clean label’ trend, rising drug resistance, holistic wellbeing approach of the post-pandemic period, and sustainable living has intensified the development of plant-derived compounds called biologically active components [1,2,3]. Biologically active substances bind by interaction or binding to specific receptors in stem cells, improving a particular physiological function of the body. Unfortunately, many such compounds are present in cytosolic cell spaces and plant cell walls [4]. Many extraction methods cannot achieve these compounds and thus obtain the highest components yields. That is why enzymes incorporation in various extractions is currently one of the few methods to provide this result. Enzymes with specific hydrolytic properties are used to degrade this matrix to gain access to biologically active components from cytosolic spaces and cell walls [5]. Nevertheless, the global market for industrial enzymes is expected to grow up to $9.2 billion by 2027 [6]. One of the advantages of the usage of enzymes is that they can be added to hydrophilic and multi-step lipophilic extractions, especially for by-product valorization [7]. For example, in Europe, grain, fruit, and vegetable food loss from post-harvest to distribution varies from 20, 41, and 46%, respectively [7,8]. However, enzyme-assisted incorporation increases phenolic content in lipophilic extracts, which is potently applicable for nutraceuticals or pharmaceuticals. However, limitations in the safety of by-products have risen, and greater attention is given to this topic [8]. In comparison, for hydrophilic extracts, enzymes efficiently increase the water-soluble content of novel derivatives applicable in food industries [9].

Due to the high demand for diverse health outcomes, functional food categories arise during the post-pandemic period, especially probiotic and prebiotic categories [10]. Incorporating these foods into human diets may reduce obesity. According to WHO, from 2018 to 2030, obese children will reach from 150 billion to 250 billion, respectively [11]. Gut microbiota modulates lipogenesis and cholesterol synthesis. Dysbiosis initiates higher absorption of sugars in the small intestines by modulating membrane transport [11,12]. Moreover, acetate, the metabolite made by the gut microbiome in the proper amount, can boost immune responses by promoting B10 cells, and in higher amounts can lead to adiposity [13,14,15]. These challenges invite scientists to search for sustainable and functional food development worldwide. One scope is enzymes, usually used for plant-based drinks production and syrups for saccharification, decreased viscosity, higher yield, and low toxicity in the food industry. However, lately, studies suggest that controlled enzyme-assisted extraction could lead to a higher and broader density of nutrients [16,17]. For example, dietary fibers with three or more monomeric units, phenolic compounds, and complexes can be suggested prebiotics and used for functional food development [18]. Chen et al. [19] investigated amylolytic and cellulolytic enzymes impact of releasing phenolic compounds and the correlation in solid-state fermentation with significant results for increased phenolic compounds content and antioxidant activities [19]. 

Because the plant material is complex, with varied compositions and matrices, enzymes are used in mixtures or cocktails. Besides releasing secondary metabolites and small peptides, they cleave long-chain molecules into shorter ones. Likewise, these substances are soluble in the solvent and can enhance organoleptic, technological, and functional properties. Moreover, enzymatic extraction methods are characterized by mild reaction conditions, substrate specificity, industrial applicability, and many other advantages [20]. These extracts may be used continuously in many fields and, surprisingly, in green synthesis development [16,21,22]. 

Green nanoparticle synthesis in aqueous plant extracts has increased over the last decade. Scientific discussion and research indicate the appropriate size of nanoparticles with high potential antimicrobial properties, involving the most common pathogenic bacteria like *Escherichia coli*, *Staphylococcus aureus*, and widely spread, highly resistant *Candida albicans* [23,24]. Studies identify that phenolic compounds and sugars play an essential capping and stabilizing role in green nanoparticles synthesis, and enzymes incorporation could increase the synthesized media yield with economically friendly conditions [25,26].

This review briefly suggests scientific approaches of commercially used hydrolases and carbohydrases for various plant materials to extract functional ingredients, products development, and possible applications.

## 2. Carbohydrases and Phenolic Compounds in Plants

**Figure 1:**
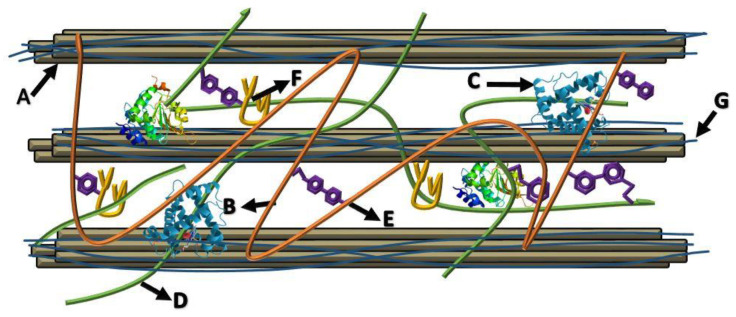
Plant cell wall graphical scheme, describing cross-linked phenolic compounds, peptides, and polysaccharides network adapted from Acosta et al., 2014 and Carpita et al., 2020 [29,30]. A—cellulose from cellulose microfibrils, B—hemicelluloses consisting of xyloglucans, glucuronoarabinoxylan, (1–3) (1–4) β glucans and glucomannan. C—structural proteins, D—pectin consisting of homogalacturonan, xylogalacturonan, and rhamnogalacturonans I and II; E—phenolic compounds, F—lignin; G—xylan and mannan coating of cellulose microfibrils.

In this review, carbohydrases and hydrolases get a more profound overview due to the plant cell wall mainly consisting of various carbohydrates, trapping active biological components. The cell contains various linear heterogeneous polymeric carbohydrates homologous to cellulose, such as xyloglucans and mannan, and hemicellulose is covalently linked to cellulose microfibrils and lignin to form complex structural branches. This multi-component structure in the plant cell wall is called lignocellulose. However, plant polymeric substances are usually categorized to waste. The global agricultural sector is estimated to produce 5 × 10^9^ tons of plant-derived biomass each year, where the total amount of lignocellulosic waste is about 2 × 10^11^ tons per year [27,28]. The structure of lignocellulose gives the stability and resistance of the cell to the extraction of internal cellular components (Figure 1), where various enzymatic activities are required to degrade all the different forms of hemicellulose.

Enzymes are derived from bacteria, fungi, yeasts, archaea, animal organs, or plant extracts. However, microbial enzymes are more stable compared to ones having a plant or animal origin. Moreover, the production of the enzyme during microbial fermentation is cost-effective and easily adapted to modifications and high purity [31]. Carbohydrases can be categorized in starch-degrading enzymes: amylases and glucoamylases; and non-starch polysaccharides (NSP) catalyzing enzymes with cellulolytic, pectinolytic xylanolytic activities [31,32,33]. In general, NSP enzymes also can be named xylases, cellulases, and pectinases due to being composed of glycoside hydrolases, carbohydrate esterases, xylanases, etc. (Table 1).

NSP enzymes are preferred as a part of commercial enzyme mixture, thus ensuring complete lysis of cell walls while contributing to a cost-effective means [5,34]. Various fungi, including *Trichoderma* sp. and *Aspergillus* sp., produce carbohydrate-hydrolyzing enzymes. For many years, *Trichoderma* sp. has been extensively studied for high cellulase production [35]. However, most strains of *Trichoderma* are known to have low β-glucosidase activity, which causes cellobiose accumulation. Although much effort has been made to obtain *T. reesei* mutants by classical mutagenicity, such as RUT-C30, the relatively low activity of β-glucosidase remains one of the significant barriers to efficient cellulose hydrolysis [36]. *Aspergillus* sp. is important in xylanase production, and the latest studies showed UV-irradiated *Aspergillus* mutants for a higher yield of enzymes [37,38]. Endoxynalases, specifically endo-β-1,4-xynalases, are the most important, depolymerizing xylan polymer into small branches. Xylooligosaccharides later are converted to xylotriose, xylobiose, and xylose [33]. Another essential component of the cell wall is pectin. It is a polymer of α-D-galacturonate and L-rhamnose units linked to α-1,4 or 1,2 to form so-called pectic elbows. Pectin, associated with cellulose, imparts stiffness and cohesion to cell walls [5,34]. Galactose, mannose, fucose, arabinose, xylose, and L-methyl, O-acetyl groups all these components make four main regions of pectin structure: rhamnogalacturonan I (RG-I), rhamnogalacturonan II (RG-II), homogalacturonan (HG), and xylogalacturonan (XG), which are involved in reducing inflammatory processes in human [39]. Pectinolytic enzymes or pectinases were the first enzymes commercially available in wine and fruit juices, although the cell wall structure was only determined later [31]. They consist of three main classes: protopectinases, esterases, and depolymerases. Protopectinases occur naturally and are responsible for dissolving otherwise undissolved protopectins from immature fruit during maturation. Esterases or pectin methylesterases remove esterified units to remove methoxy esters. Depolymerases are represented by lyses and hydrolases that catalyze the fragmentation of glycosidic bonds. Today, pectinases are used in the fruit juice industry because of their effectiveness: higher juice yield, filterability, lower viscosity, and increased transparency. Pectinolytic enzymes have been found to be particularly effective in the extraction of polyphenols, particularly in the release of anthocyanidins from glycosides. Many pectinolytic mixtures sold today contain all three types of the above classes and a mixture with cellulases and hemicellulases to achieve an overall synergistic effect [5,40,41]. 

In general, biological raw material systems range from 5000 to 25,000 individual phytochemicals that can have biological activity. Biologically active substances are metabolites synthesized in plants that perform plant protection and other functions. There is growing evidence that biologically active substances can help maintain optimal health and reduce the risk of chronic diseases such as cancer, cardiovascular disease, stroke, and Alzheimer’s disease (AD) [42,43]. For example, quercetin found in pines, buckwheats, and many other plants has a high binding link through hydrogen bonds to the cyclin-dependent kinase 6 (CDK6) and inhibits its activity, which plays an essential role in the progression of different types of cancer [44]. However, phenolic compounds are usually soluble conjugates (glycosides) or insoluble forms (phenolic acids) covalently linked to carbohydrate radicals or cell wall structural components such as cellulose, hemicellulose, lignin, and pectin. In the insoluble form, the phenolic compounds are linked to the structural cell wall components by covalent bonds. Phenolic acids bind to lignin through the hydroxyl groups of their aromatic rings and various polysaccharides and other proteins through ester linkages. They perform a protective function. Significant interests are given to flavonoids which are found in the form of glycosides bound to sugar residues via –OH groups (O-glycosides) or via –C–C– bonds (C-glycosides) [44,45]. The latest article discovers the promising neuroprotective and memory-enhancer characteristics of flavonoid-rich food and plant extracts by increasing the functions of neurons and cell proliferation [46]. Flavonoids are involved in regulating kinase-signaling cascades, including PI3K/Akt, PKC, and MAPK pathways [46,47]. In the food industry, functional food components are used to replace preservatives due to their antioxidant and antimicrobial properties [48,49,50]. Unfortunately, solvent-based extraction of biologically active substances often suffers from low extraction efficiency, requires a long extraction time, and often leaves traces of organic solvents in the final product, which reduces the quality of the product [51,52]. Therefore, there is a need to develop optimized, step-by-step extraction methods for beneficial substances for each type of raw material. Therefore, detailed protocols are required to produce bioactive compounds, especially from plants where the cell wall may inhibit the extraction efficiency [7,49]. Enzymatic extraction of biologically active compounds from plants is a potential alternative to conventional solvent-based extraction methods. Enzymes are ideal catalysts for extracting, modifying, or synthesizing complex biologically active compounds of natural origin. Enzyme-assisted extraction is based on the inherent ability of enzymes to catalyze reactions with exceptional specificity and the ability to function under mild processing conditions in aqueous solutions [53]. However, parameters such as enzyme specificity, activity, botanical origin, pH, enzyme and substrate, liquid to solid ratios, temperature, and time are essential for obtaining the highest value results [54,55].

Carbohydrases-active enzymes database (CAZy; https://www.cazy.org/, accessed on 10 February 2022) classified five main classes: glycoside hydrolases, glycosyltransferases, polysaccharides lyases, carbohydrate esterases, and auxiliary activities [56]. A mixture of these enzymes has to be implemented into reactions on the results maintained to achieve. Ideal selected enzymes have high activity and regio-/stereo-selectivity [57]. Moreover, raw materials of plant origin consist of a complex system of various macro-/micro-components [29]. Plant origin, morphological part, cultivar, and growing conditions impact the storage of secondary metabolites. The approach of Woo et al. [58] of fifteen cultivars in oats showed the variation by cultivars and harvesting days of phenolic content from 3748.4 to 5700.0 mg/100 g [58]. Enzyme usage usually requires low temperatures, usually 50–60 °C; short extraction periods of up to several hours, waste-free production possibilities, reduced substrate specificity, allowing extraction of many bioactive compounds, otherwise, were made inaccessible. [45,59]. Examples of various plant material extraction are presented in Table 2. Additionally, the yield of the extract is often characterized by high quality and bioavailability. Gonzalez et al. [60] compared ultrasound-assisted extraction with enzyme-assisted extraction for anthocyanins from blackcurrants, where a significant difference was not evaluated. Major two Cyanidin 3–O–rutinoside (C3R) and Cyanidin 3–O–glucoside were extracted at similar quantities and accounted for up to 90%. C3R stimulates the mechanism of insulin secretion through INS-pancreatic β-cells by promoting calcium channels and activating the PLC-IP3 pathway [61].

Furthermore, the method also allows applying greener chemistry in the food industry and pharmaceutical companies to optimize purer ways to extract new compounds. Enzymes can disrupt specific bonds and interactions in cell walls and membranes, resulting in higher extraction yields of bioactive substances [40,67,68]. 

## 3. Enzymatically Treated Polysaccharides as Possible Functional Components

**Figure 2:**
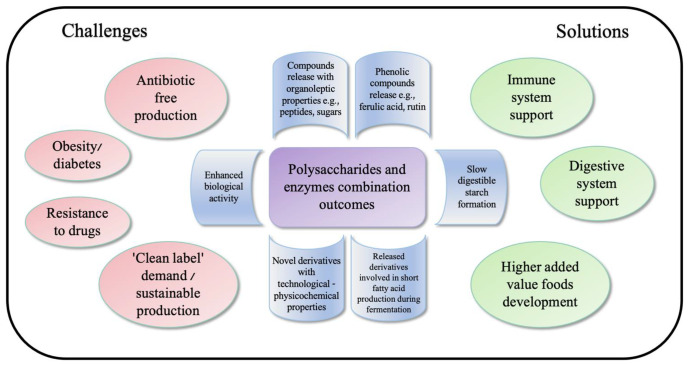
Summary graphic of polysaccharides and enzyme combination outcomes connected to solutions for uprising challenges.

Lignocellulose and starch content differs depending on plant material. Grain materials usually consist of more starches because of the endosperm in the seed, whereas other plant parts, like brans, leaves, and others, consist of non-starch polysaccharides. Shah et al. [62] described three categories of starches: rapidly digestible starch (RDS), resistant starch (RS), and slowly digestible starch (SDS), which relate glucose release during digestion. Mainly, starches are found in cereals and pseudocereals where β-glucans are the most widely investigated non-starch polysaccharide and have enormous health-promoting properties [69]. However, β-glucans properties may vary depending on molar mass, which can be from 209 up to 2500 (kg mol^−1^) depending on the cultivar, variety, and the location of growth [70,71]. Nonetheless, β-glucans are found in fungi, yeasts, bacteria, and seaweed. The main difference is the ratio, length, and linkage between D-glucose of β-(1,3) and β-(1,4) for cereal grains β-(1,3) and β-(1,6) for fungi and yeasts [72]. For oats, novel oligosaccharides were produced by hydrolyzing β-glucans with dual enzyme-assisted hydrolysis using α-amylases and transglucosidase in result shorter α-(1,6)-branch linkage glucans, which stabilize the glucose release during the intestinal phase in vitro and may be prebiotic [73]. Another study in vitro also resulted in SDS increasing with corn starch using branching enzymes and maltogenic alpha-amylase [74]. Moreover, as a major non-starch polysaccharide in cereal grains, arabinoxylans are getting attention for their prebiotic properties, where enzymatically hydrolyzed fiber releases ferulic acid or combines with arabinose [75,76]. In general, dietary fibers, with more than three monomeric units known as prebiotics, are a growing trend for immune support through the microbiome and may prevent the uprising resistance to drugs for humans and animals [10]. Moreover, because antibiotic-free products are getting more attention from consumers, feed enhancement with dietary fibers may support the immune system of animals [19,75]. However, the clinical study by Wastyk et al. [77] for identifying humans‘ health marker variation while having the intervention of 10 weeks of high-fiber diet showed an increase in short-chain fatty acid (SCFAs), the density of microbes for higher protein content, and quantity but no diversity of microbiome [77]. It suggests that microbiota was insufficient to process increased fiber intake, even though glycan-degrading carbohydrate-active enzymes increased [77,78]. These studies implement the essential role of carbohydrate lengths for human diets. Animal studies with rats showed the xylan-oligosaccharides (XOS) as a potent dietary supplement of obesity prevalence. XOS promotes growth of *Bifidobacterium* strains involved in the development of obesity and insulin resistance [14,79]. In between, galacto-oligosaccharides (GOS) in studies with rats implement the potency of gut recoveries after alcohol withdrawal by a significant increase of butyric and propionic acid and the proliferation of *Lactobacillus* and *Bifidobacterium* strains [80]. Another animal study with GOS extracted from mulberry treated with β-mannanases showed that 200 mg/kg/day can initiate expression of crucial glycolysis enzymes such as GK, PK, and PCB and proteins p110 and Akt of key signaling intermediates, which results in the prevalence of diabetes and obesity [81]. A graphical scheme of summarized polysaccharides and enzymes combination results is presented in Figure 2. 

## 4. Enzymes-Assisted Processes for Plant Materials

### 4.1. Bioactives Extraction from By-Products

In order to use the enzyme or their mixtures efficiently in extraction methods, it is essential to understand their catalytic mechanism of action and the optimal activity conditions for the recovery of individual biological raw materials and substances: e.g., a mixture of cellulose, pectin, and hemicellulose enzymes in a grapefruit peel during hydrolysis releases sugar into monomeric compounds that microorganisms can use later to produce ethanol and other fermentation products [82]. Another example is in tomatoes: lycopene is found mainly in the peel, giving it a red color. Carotenoids, especially lycopene, are one of the most potent antioxidants of plant origin, with a role in more than twenty different induced-signaling pathways and cell cycles described by Qi et al. [83]. According to scientific knowledge, lycopene is better absorbed from processed products than fresh tomatoes [84,85]. The digestive enzyme pancreatin is recommended before the solvent extraction of lycopene. Its use increased the yield of lycopene 2.5-fold compared to that obtained using the traditional extraction method [54]. Using Pectinex Ultra SP–L (P, pectinolytic enzyme), Celluclast (C, cellulolytic enzyme), and Viscozyme L commercial enzymes for solvent extractions, the yield of lycopene was increased as well as antioxidant activity compared with samples without enzyme mixtures [84]. Moreover, the scientific literature shows that in the stepwise extraction of buckwheat husk using xylanase commercial preparations, the yield of soluble fractions increased 4–5 times compared to the control extraction [7]. The use of enzymes reduces the amount of solvent required for extraction and increases the yield of extractable compounds. For example, tannase, pectase, cellulase, and hemicellulase are widely used in juices to increase product yield and improve quality [40,86]. However, when it is enzymatic extraction alone, compare with other extraction methods. Zheng et al. [87] described different extraction methods of palm kernel expeller. Dried extracts prepared with enzymes, hydrochloric acid, carboxymethylation, and hydroxipropylation were compared among chemical composition and physicochemical and functional properties. All the extract methods increased dietary content, phenolic content, and physicochemical properties. However, the goals of extract application have to be implemented because all extract methods were suitable, although enzyme assistance required fewer organic solvents, which implements in sustainability coverage [87].

Enzyme-assisted extraction (EAE) presents applicability to extract pectins from wastes and by–products by increasing plant cell wall permeability [88,89]. Enzymes are applicable to extract many phenolic compounds, including flavonoids and anthocyanidins [90]. Enzyme activity, treatment time, substrate ratio, and particle size are essential to get the highest efficiency during enzymatic treatment. Optimized conditions were discussed for pistachio green hull extraction by Yazdi et al. [84]. This research used cellulases, pectinases, tannases, and their mixtures for the extraction. Results indicated that all of the three enzymes at the same time used to extract phenolics were giving the highest score [91]. Moreover, by-products may be applied as enzymes production during solid-state fermentation (SSF). Some of the highest by-products of cacao are bean shell, brewers’ spent grain, and wheat bran, which have an estimated production of 140 million tons per year. These were implemented for SSF with *Aspergillus awamori*, *Aspergillus niger*, and *Aspergillus oryzae*, which produce feruloyl-esterases and amylases. These enzymes in bread enhanced ferulic acid quantity, total phenolic content, and antioxidant activity [92,93].

### 4.2. Plant-Based Drinks from Grains and Fermented Drinks Production 

**Figure 3:**
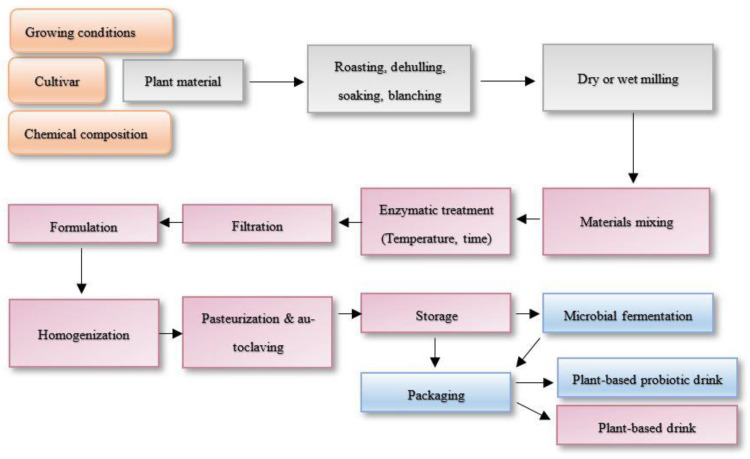
Chart flow of plant-based prebiotic and probiotic drinks production.

There is also no surprise that dairy milk substitute from grains production often requires enzymatic assistance for increasing extraction yields, proteins, and total solids content [22]. Annually, global plant-based dairy substitutes were marked to be grown by 10% and by 2019 had reached US 1.8 billion dollars [94]. Moreover, created derivatives and released sugars create sensory-acceptant organoleptic properties [16]. Amylolytic enzymes are required due to the amylose and amylolytic ratio of the starches, resulting in different rheological and textural properties. However, disrupting amylose and amylopectin molecules increases liquefying properties of grain beverages. Many starch-modifying enzymes including α-amylase (EC 3.2.1.1), β-amylase (EC 3.2.1.2), glucoamylase (EC 3.2.1.3), α-glucosidase (EC 3.2.1.20), pullulanase or amylopullulanase (EC 3.2.1.41), and cyclodextrin glycosyltransferase (EC 2.4.1.19) are used in industries that cleave the α-1,4- or α-1,6-glycosidic linkages, leading to the release of reducing sugars and oligosaccharides which are possibly prebiotic [32,95]. Interestingly, a quantitative prebiotic score of plant-based dairy substitutes can be identified, describing which prebiotics foster probiotic strains’ selective growth. Phenolic compounds, reduced sugars, and oligosaccharides are also great nutrients for bacterial growth, and the fermented food sector is rising due to their possible probiotic functions [66,96]. However, different plant materials are different sources for particular strains [76,97]. Enzymes are widely applicable and potent as precursors of nutrient production and initiate the growth of fermentation starter cultures: e.g., *Lactobacillus* strains *L. reuteri* L45, *L. plantarum* L47, and *L. johnsonii* L63 growth were initiated by cellulase- and pectinase-degraded rapeseed fibers [98]. Nissen et al. [99] identified prebiotic scores with different selectivity of hemp, soy, rice, and their mixtures, where hemp and soy drinks initiated the highest growth of *L. plantarum* 98b, and for growth *B. bifidum* B700795 mixture of hemp-rice and hemp-soy drinks, showed the potent scores [99,100]. Five main steps are incorporated in the flow chart for manufacturing enzymatically treated and by-the-step fermented plant-based drinks from plant materials: plant disruption, extraction, formulation, fermentation, and packaging [94]. However, it is also important not to forget the cultivar’s selectivity to get the highest value product [101]. Figure 3 represents the summarized flow chart of plant-based drinks production, where additional importance is given for plant material selection due to European Commission strategy incorporation ‘from farm to fork’, which is the heart of the European Green Deal priorities from 2019–2024. Continuing on, the flow chart represents the prebiotic plant-based drink production, which further on leads to probiotic drink production which can be mono- or multi-microbial [94]. Shori et al. [102] reviewed the plant-based dairy substitutes fermented with probiotic strains functionality, shelf-life expansions, and nutrition value enhancement [102]. 

Moreover, plant-based fermented products have properties against pathogenic bacteria, and pH is lower than regular plant-based drinks, affecting product stability [103]. However, another aspect of ingested probiotic food pathway and viability may be investigated. The texture and nutrient density and the complexity of the food matrix are responsible for releasing the nutrients delivering specific metabolites and strains to the intestines [104,105]. Moreover, the density and variety of live cultures may be the essential indicators for increasing the variety of gut microbiome [77]. 

### 4.3. Nanocrystals, Nanofibers, and Nanocellulose

The latest studies indicate that phenolic content increased in fermented products [96,106,107]. As a sidestream nanocellulosic material, it is usually produced by *Komagataeibacater, Acetobacter, Gluconacetobacter* strains which might be used as, e.g., wound healing biofilm [108]. Specific enzymes release, cleave, transport, and form derivatives from different plant origins by opening the ability to discover green synthesis applications for nanofibers, nanocrystals, and nanoparticles. Aqueous different plant extracts are the new scientific approach for synthesizing nanoparticles by changing environmentally disruptive chemical and physical methods. Enzyme-assistance by disrupting plant cell wall microfibrils and amorphous zones is visible through Transmission Electron Microscopy (TEM), Atomic Force Microscopy (AFM), or Scanning Electron Microscopy (SEM), which also implies an increase of extract yield [109,110]. Plant extracts contain high phenolic content and reducing sugars and reducing or stabilizing agents [26,111]. Additionally, as mentioned before, increasing drug resistance and nanofibers formation is getting attention for possible antibacterial and drug delivery properties and nanocellulose formation for biodegradability, non-toxicity, and potential physicochemical properties [112]. Yarbrough et at. [113] described cellulolytic enzymes performance of nanofibrils and nanocrystals formation by depolymerizing carbohydrates into smaller units [113]. Depending on the plant origin, charges and forces change using specific cellulolytic activity characterized enzymes [68]. 

**Figure 4:**
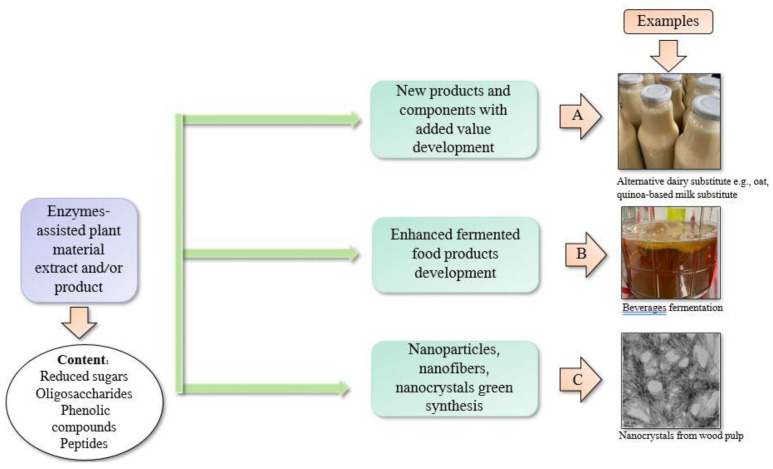
Enzyme-assisted extract or possible product applications, where A presents alternative dairy substitutes development [22], B—fermented beverages production [96], C—nanocrystals formation from enzymatically treated wood pulp [114]. A and B photographs were taken by the authors at the Lithuanian Research Centre for Agriculture and Forestry.

Summarized application of enzymes-assisted extraction and their products is described in Figure 4.

## 5. Existing Rules on the Use of Enzymes and Their Products

At present, in the European Union, food enzymes usage is described under Regulation (EC) No 1332/2008 of the European Parliament and of the Council of 16 December 2008 on food enzymes. Enzymes are considered food additives because they are found in the final products only in inactivated form and in smaller amounts. The definition of food enzymes are described in Regulation (EC) No 1169/2011 [34]. Specifically, directive 2000/13/EC describes labeling requirements for food enzymes. However, some limitations occur while extracting by-products: first, the lack of specific regulations to assure the safety of valorized products. For human consumption, some substances of food by-products are categorized as food additives and described in EC Regulation No. 1881/2006. However, it is categorized as nutraceutical; these products are covered by food supplement Directive 200/46/EC. Moreover, the contamination of food by-products must be implemented, which is described in Council Regulation 315/93/EEC. Secondly, if by-products are enzymatically modified, EC regulation No 258/97 (1997) could apply to food and food ingredients with a new or intentionally modified primary molecule structure if not used before May 1997. This regulation ensures the safety assessment before entering the EU market. While entering the USA market, Food and Drug Administration (FDA) regulates food additives (Sec. 348), new dietary ingredients (Sec. 350b), pesticide chemical residues (Sec. 346a), and others through Federal Food, Drug, and Cosmetic Act (Chapter 9, Subchapter IV) [8]. Moreover, for worldwide fair food trade, international food standard CODEX Alimentarius, which is covered by the World Health Organization (WHO) and Food and Agriculture Organization of the United Nations (FAO), also describes food enzymes as food additives. Depending on how a product is made, the usage is characterized by enzyme name, the producent, and the highest dosage. In general, for the standard of food additives CODEX STAN 192-1995 for flours and starch products, two enzymes are described. In detail, for α-amylase and glucoamylase, whose producers are *Bacillus subtilis* (INS 1100(iii)) and *Aspergillus oryzae* var. (INS 1100(iii)), dosage is not identified. However, all food additives, including food enzymes such as xylanases, pectinases, and others, are collected in the list of CODEX Specification for Food Additives CXN 6–2019 [115]. However, for enzymes producers, the recommended purity specifications for food-grade enzymes are given by the Joint FAO/WHO Expert Committee on Food Additives (JECFA) and the Food Chemical Codex (FCC). 

## 6. Conclusions

The use of enzymes in extracting biological raw material compounds is an up-and-coming area from small-scale, laboratory optimization studies to large-scale, industrial applications. It implies food processing, functional components, and medical devices development for high antioxidant, anti-inflammatory, and antimicrobial characteristics. However, success in this area requires interdisciplinary research from various life sciences disciplines. An important area of research is investigating the stability of enzymes and their interaction with other food and plant ingredients during processing and storage; repeatability is also questionable because the plant material differs from the origin, cultivars, and growing, harvesting, and storage conditions. Additionally, limitations occur in the form of worldwide regulations of enzymes usage and dosages due to the novel components that are produced during these processes. However, enzyme-assisted processes are reaching for more sustainable development of innovations in a broad spectrum of industries. 

## Figures and Tables

**Table 1:** Structure of commonly found carbohydrates in plant material and enzymes usage of their cleavage [30,31,32,33].

	Non-Starch Polysaccharides		
	Hemicellulose	Cellulose	Pectin
Consist of	xyloglucans	cellulose nanofibrils:	homogalacturonan
	glucuronoarabinoxylan	(a) xylan	rhamnogalacturonan I and II
	β–glucan	(b) mannan	xylogalacturonan
	glucomannan		
Enzymes used in processes	Xylanases:	Cellulases:	Pectinases:
	exoxylanases	endo–(1,4)–β–d–glucanase (EC 3.2.1.4)	polygalacturonases
	β–xylosidases,	exo–(1,4)–β–d–glucanase (EC 3.2.1.91)	pectin esterases
	xylan–1,4–β-xylosidase	β–glucosidases (EC 3.3.1.21)	pectate lyase
	endoxylanases	β–glucosidases (EC 3.3.1.21)	
	**Starches**		
Consist of	amylose		
	amylopectin		
Enzymes used in processes	α–Amylases (EC 3.2.1.1)		
	β–amylase (EC 3.2.1.2)		
	glucoamylase (EC 3.2.1.3)		
	α-glucosidase (EC 3.2.1.20)		
	pullulanase or amylopullulanase (EC 3.2.1.41)		
	cyclodextrin glycosyltransferase (EC 2.4.1.19)		

**Table 2:** Examples of various substrates technological parameters for enzyme-assisted extraction.

Enzymes	Producent	Substrate	Enzymes Quantity	Liquid to Substrate Ratio	pH	Temp. °C	Time	Ref.
Xylanase cocktail	*A. niger*	Citrus fiber	0.45%	1:20	4.5−6.5	50	120 min	Song et al. [62]
Cellulase	*A. niger*	Coffee by-products	5−15 U	1:25	5.0−6.0	50	30−20 min	Belmiro et al. [63]
Cellulase from Celluclast 1.5 L	*T. reesei*	Banana peel	5 FPU/ml	1:20	6.0−7.0	50	120 h	Phirom-on et al. [64]
Pectinase	*A. niger*	Guava pulp	0.10%	2:5	2.97−3.97	45	3–90 min	Ninga et al. [65]
Pectinase	*A. niger*	Blackcurrant	108 U/g	0.1:15 and 0.2:15	5−6	60	10−90 min	Gonzalez et al. [60]
Heat stable alpha-amylase	*Bacillus sp.*	Oat flours	0.01%	1:5	5.0−9.0	100	15−75 min	Chen et al. [66]

## Data Availability

The data presented in this study are available in the article.
